# Bagaza virus is pathogenic and transmitted by direct contact in experimentally infected partridges, but is not infectious in house sparrows and adult mice

**DOI:** 10.1186/s13567-015-0233-9

**Published:** 2015-09-04

**Authors:** Francisco Llorente, Elisa Pérez-Ramírez, Jovita Fernández-Pinero, Maia Elizalde, Jordi Figuerola, Ramón C. Soriguer, Miguel Ángel Jiménez-Clavero

**Affiliations:** Centro de Investigación en Sanidad Animal, Instituto Nacional de Investigación y Tecnología Agraria y Alimentaria (INIA-CISA), Ctra Algete-El Casar s/n, Valdeolmos, Spain; Estación Biológica de Doñana (EBD-CSIC), Avenida de Americo Vespucio s/n, Seville, Spain

## Abstract

Bagaza virus (BAGV) is a mosquito-borne flavivirus belonging to the Ntaya serocomplex. In 2010, a disease outbreak was reported in Cádiz (Southern Spain) affecting game birds (red-legged partridges and common pheasants). In this work, red-legged partridges were inoculated experimentally with infectious BAGV isolated from this outbreak in order to make a complete clinical and analytical assessment of the disease caused by the pathogen in this species. Viral load (by real-time RT-PCR) in blood, oral and cloacal swabs, and feathers, and neutralizing antibody titres (by VNT) were measured. In order to determine direct contact transmission, non-inoculated partridges were caged together with the inoculated ones. To assess infectiousness in other species, house sparrows and mice were also inoculated with the virus. All the inoculated partridges were clinically affected, and 30% of them died. All the infected individuals lost weight, with larger losses being recorded in females. Conversely, no mortality or disease symptoms were observed in the sparrows or mice. Remarkably, all the contact partridges acquired the infection by direct (non-vectored) transmission. This study confirms that the red-legged partridge is a susceptible host for BAGV infection, and that this pathogen is transmitted by direct contact. Long-lasting viral loads detected in calami of immature feathers demonstrate that feather sampling could be a useful strategy in active surveillance programs for early detection of BAGV.

**Electronic supplementary material:**

The online version of this article (doi:10.1186/s13567-015-0233-9) contains supplementary material, which is available to authorized users.

## Introduction

The incidence and geographical distribution of mosquito-borne epornitic flaviviruses have increased in the last decade in different parts of the world [1]. Europe and the Mediterranean Basin constitute good examples of the recent introduction and geographical spread of these types of flaviviruses, which are of concern for both animal and human health, e.g. West Nile and Usutu viruses [1-4]. Likewise, other flaviviruses have also appeared recently beyond their known geographic ranges. This is the case of Bagaza Virus (BAGV), belonging to the Ntaya serocomplex, in Southern Spain in September 2010, associated with an unusually high mortality of red-legged partridges (*Alectoris rufa*) and ring-necked pheasants (*Phasianus colchicus*) [5]. This outbreak was the first one of BAGV detected in Europe, and enabled its first isolation from a vertebrate host. Although no clinical disease has been reported since the outbreak in 2010, specific Bagaza virus-neutralizing antibodies were detected in 2011–2012 in shot birds from both species in the same region, including juvenile partridges suggesting continued circulation of the virus [6].

BAGV was first isolated in the Central African Republic in 1966, from a pool of *Culex* mosquitoes [7]. Subsequently, it was detected in mosquitoes in other countries in Western Africa [8-10] and India [11]. A comparison of full-length sequences indicates that BAGV and Israel turkey meningoencephalomyelitis virus (ITV), a pathogen affecting turkeys reported in Israel and South Africa, are the same virus species, and the name Avian meningoencephalomyelitis virus (AMEV) was proposed to unify them [12].

Information on the pathogenesis of BAGV has recently been obtained from natural Bagaza infection during the outbreak in Southern Spain in 2010 [13-15]. As regards experimental infections, previous studies have been mainly limited to old ITV and BAGV strains inoculated in domestic turkeys [16] and mice [17], while experimental inoculations of recent BAGV strains are lacking. Therefore, the first aim of this study was to assess if a BAGV isolate from a recent European outbreak is able to reproduce, under experimental conditions, the disease observed in the field in the same bird species from which it was isolated.

Hence, the course of the viremia, seroconversion, viral distribution in organs/tissues and virus shedding through different routes were examined in red-legged partridges in order to gain knowledge about BAGV pathogenicity and tissue tropism in this species, and its role as a reservoir host. Possible contact transmission and the usefulness of different samples for diagnosis were also studied. Moreover, the same BAGV isolate was inoculated in the house sparrow (*Passer domesticus*) and in mice (*Mus musculus*) in order to assess its infectiousness and pathogenicity in these species and to further assess the host range for this virus.

## Materials and methods

### Virus isolation and preparation of inocula

The virus used in the inoculations was isolated from the heart of a partridge found dead in the outbreak registered in Southern Spain in 2010 [2]. The original tissue (heart) was homogenized, clarified and inoculated in BSR cells (clone of BHK-21 cell line). After two passages in BSR and four passages in Vero cells, a cytopathic effect was observed in the cell cultures, from which the virus was recovered, grown and titrated by plaque assay in Vero cells as previously described [18]. The isolated virus, named *BAGV Spain RLP-Hcc2/2010*, was completely sequenced [GenBank:KR108245]).

### Birds and mice

#### Red-legged partridges

Five-month-old red-legged partridges (*Alectoris rufa*) (*n* = 30) were obtained from the Lugar Nuevo red-legged partridge breeding facility (Estación de Referencia de la Perdiz Roja, Consejería de Medio Ambiente y Ordenación del Territorio-Junta de Andalucía, Andujar, Spain, 38°16′N 4°6′W), which guarantees the lack of hybridization with other partridge species. Prior to the experiment, all individuals were tested serologically using a commercially available competitive ELISA (Ingezym West Nile Compac, INGENASA, Madrid, Spain) and virologically (by real-time RT-PCR as described below) to ensure that previous exposure to BAGV or other flaviviruses had not occurred. The partridges were transported to the biosafety level 3 (BSL-3) facilities at CISA (Centro de Investigación en Sanidad Animal, Valdeolmos, Spain). After careful deparasiting, the birds were distributed in three wire mesh cages (120 × 40 × 40 cm) in experimental groups, (see below this section, under “Experimental inoculation”), composed of 50% males and 50% females (estimated considering body size and presence of spur, and confirmed after necropsy at the end of the experiment). The birds were provided with a commercial diet for game birds and water *ad libitum* throughout the experiment.

#### House sparrows

Free-living house sparrows (*Passer domesticus*) were captured using mist nets and banded in Southern Spain (Seville) and adapted to captivity for two months in outdoor cages. The sparrows were transported to the BSL-3 facilities at CISA, deparasited and housed in individual wire cages (45 × 40 × 30 cm) and tested serologically and virologically to discard previous exposure to flavivirus, as explained above for red-legged partridges. The sparrows were provided with mixed bird food and water *ad libitum*.

#### Mice

Three-week-old Swiss HSD ICR (CD1) outbred female mice were purchased from Harlan Inc. (Switzerland) and kept in the CISA BSL-3 facility until their inoculation at the age of 4 weeks.

Animal care, handling and experimental procedures were authorized by the INIA Committee of Ethics and Animal Experimentation (Reference codes: BV/Pe-1, BV/Go-1 and BV/Mo-1) according to European and Spanish laws on the protection of animals for experimental and other scientific purposes (Spanish Royal Decree 53/2013 and Council Directive 2010/63/EU).

### Experimental inoculations

After 7 days for acclimatization, the birds and mice were inoculated with BAGV (strain Spain RLP-Hcc1/2010) diluted in up to 0.1 mL in Dulbecco’s Minimum Essential Medium (DMEM) (supplemented with 2 mM L-glutamine, 100 U/mL penicillin and 100 μg/mL streptomycin).

Distribution of animals in experimental groups: One group composed of 10 red-legged partridges (inoculated group) and another group of 6 birds (programmed necropsy group) were inoculated subcutaneously in the neck with approximately 2 × 10^5^ pfu (plaque-forming units) of BAGV/individual. Both groups were kept in separated cages. A third group, consisted of four non-inoculated partridges (contact group), was kept with the inoculated group, in the same cages. One additional group of partridges (control group; *n* = 10) was sham-inoculated with an equivalent volume of DMEM and kept in a separate cage.

Eight house sparrows were inoculated subcutaneously in the neck with approximately 2 × 10^5^ pfu/individual of BAGV whereas another group (*n* = 8) was sham-inoculated and used as control. All the sparrows were kept in individual cages.

Four groups, each consisting of two 4-week-old mice were inoculated intraperitoneally with tenfold dilution doses of BAGV ranging from 5 × 10^2^ to 5 × 10^5^ pfu/individual. The control group was composed of two mice that were sham-inoculated and housed in a different cage.

### Clinical follow-up and collection of samples

#### Red-legged partridges

Disease symptoms were observed daily for up to 15 days post-inoculation (dpi) in the inoculated and control groups and for up to 18 dpi in the contact group. To follow the viremia course, blood samples were collected at 1, 3, 5, 7, 9, 11 and 15 dpi in the inoculated and control groups and at 3, 5, 7, 9, 11, 15 and 18 dpi in the contact group. Blood samples (0.1 mL) were collected in sterile polypropylene tubes filled with 0.9 mL BA-1 diluent (Hanks M-199 salts, 0.05 M Tris, pH 7.6, 1% bovine serum albumin, 0.35 g/L of sodium bicarbonate, 100 units/mL of penicillin, 100 μg/mL of streptomycin, 1 μg/mL of amphotericin B) and stored at −70 °C until analysis. A second blood sample (0.1-0.2 mL/ individual) was taken in dry tubes and allowed to clot at 37 °C for 1 h, followed by incubation at 4 °C overnight, to obtain serum. Similarly, immature rump feathers, oropharyngeal and cloacal swabs were collected one day before inoculation, and on the same days post-infection as blood sampling, in all groups.

Swabs were placed in sterile polypropylene tubes containing 1 ml PBS, while feathers were collected in empty sterile polypropylene tubes, and both were stored at −70 °C until analysis. Following death, the partridges were necropsied within <18 h. Individuals from the programmed necropsy group were euthanized humanely by intravenous injection of embutramide (T61 ®, Intervet - Schering-Plough, Madrid, Spain) on days 4, 7 and 10 dpi (2 birds each day) and subjected to full necropsy. Similarly, survivors from the inoculated and contact groups were euthanized at 15 and 18 dpi respectively. During necropsy, tissue samples (approximately 0.1 g) from the brain, heart, kidney, spleen and liver were collected for real-time RT-PCR analysis, using single-use scalpels and forceps to avoid cross-contamination, and placed in tubes containing 0.9 mL of PBS.

#### House sparrows

The house sparrows were monitored daily for up to 14 dpi. Blood samples (0.1 mL) were collected from the jugular vein at 1, 3, 5, 7, 9 and 15 dpi for viremia determination and at the end of the experiment for antibody detection, as described above for red-legged partridges. The birds were euthanized and necropsied as described previously for partridges.

#### Mice

The mice were monitored daily for symptoms for up to 3 weeks after inoculation (when they were euthanized). Blood samples were collected at 3 dpi (for viremia determination) and at 21 dpi for antibody detection in serum.

### Viremia, BAGV genome load and complete sequence analyses

BAGV viremia was measured by standard plaque-formation assays as described [18], while the viral genome load was measured in blood by semiquantitative real-time RT-PCR, using a previously described method [19]. A Ct = 40.0 was set as cut-off to consider the samples as positives for virus detection by real-time RT-PCR. Tissues, feathers, oropharyngeal and cloacal swabs were examined using the same RNA extraction method and the above described real-time RT-PCR, but following a different preparation step depending on the type of sample. Calami were separated from feathers and placed in tubes containing 0.9 mL of PBS (one calamus per sampling day and animal). Tissues from necropsies and calami in PBS were homogenized for 2 min at 30 cycles/s using a Tissuelyser homogenizer (QIAGEN), followed by a centrifugation step at 850 × *g* 10 min to clarify homogenates. Swabs were directly clarified at 9500 *g* for 5 min. One of the tissue samples with the highest viral genome load (brain from partridge n° 7) was subjected to complete genome sequencing [GenBank:KR108246]).

### Antibody detection assays

Virus-neutralizing antibodies to BAGV in serum were detected and titrated by a virus-neutralization test (VNT) as described [20]. Neutralization titres were assigned based on the highest dilution of each serum capable of neutralizing the infection, i.e. abolishing any observable CPE in the cell monolayer.

### Statistical analysis

Comparisons of survival rates between inoculated and control groups were carried out using the Gehan-Breslow-Wilcoxon method [21]. Weight loss (% initial weight), viremia (pfu/mL), and genome load (Ct value) data between groups were compared using Student’s *t*-test for independent groups for the different sampling days.

## Results

### Pathogenicity and clinical signs

**Figure 1:**
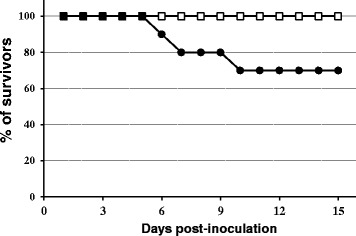
**Mortality after inoculation of BAGV in red-legged partridges.** Percentage of survivors observed at different days post-inoculation. Closed circles represent BAGV-inoculated partridges. Open squares represent sham-inoculated partridges.

**Figure 2:**
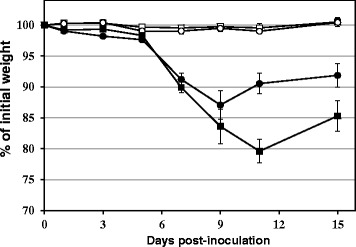
**Weight loss of red-legged partridges after inoculation with BAGV.** The course of the variation in weight in the different groups of red-legged partridges, expressed as the percentage of initial weight (i.e. weight measured on the day of inoculation), measured at different days post-inoculation. Circles indicate male and squares female. Closed symbols represent BAGV-inoculated partridges, while open symbols represent sham-inoculated partridges. Error bars represent the standard error of the mean.

The BAGV Spain RLP-Hcc2/2010 strain was pathogenic for the red-legged partridge, with an observed mortality of 30% (3 out of 10 BAGV-inoculated partridges) which occurred between 6 and 10 dpi (Table 1, Figure 1), while no morbidity or mortality was registered in the non-inoculated (control) group. However, and considering the limited number of birds by group, differences observed in survival rates between inoculated and control group were not statistically significant (*p* > 0.05). Partridges from the inoculated group showed apathy, reluctance to move, weakness, unresponsiveness and behavioral changes (e.g. lack of avoidance to capture). Some individuals also presented partially closed eyes. From 7 days post infection to the end of the experiment, all inoculated partridges showed a significant body weight loss (between 7 and 23% of initial weight) as compared to the control group (*p* < 0.00001 from 7 to 15 dpi). Indeed, female partridges suffered a significantly more pronounced and prolonged weight loss than males at 11 dpi (*p* = 0.004) and 15 dpi (*p* = 0.041) (Figure 2).

**Table 1:** **Summary of viral inoculations performed in this work**

**Species**	**Inoculum BAGV**	**Mortality rate**	**Mean viral load in blood 3dpi (Ct)**	**Ab titre range**
Partridge	2 × 10^5^	0.3 (3/10)	23.2	320 - 1280
House sparrow	2 × 10^5^	0 (0/8)	>40	<10
Mouse	5 × 10^2^	0 (0/2)	>40	<10
	5 × 10^3^	0 (0/2)	>40	<10
	5 × 10^4^	0 (0/2)	>40	<10
	5 × 10^5^	0 (0/2)	>40	10 - 20

Inoculated doses per individual are indicated as pfu; the mean viral load in blood was measured by real-time RT-PCR at 3 days post-infection (dpi) and the antibody (Ab) titre was determined by virus-neutralization test (VNT) at the end of the experiment.

In the case of inoculated sparrows and mice, no mortality or signs of disease were observed throughout the experiment (Table 1).

### Viremia

**Figure 3:**
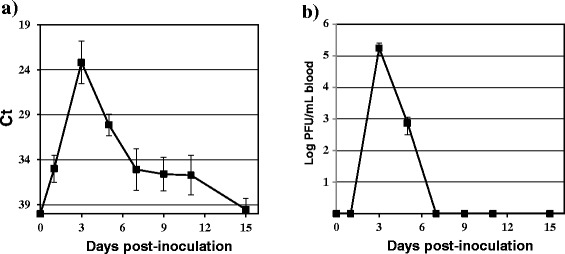
**Mean daily blood viral genome load and viremia titres for BAGV-inoculated red-legged partridges.** The course of **A** blood viral genome load and **B** viremia titres during the experiment of BAGV inoculation in red-legged partridges, determined at different days post-inoculation. Each point represents the mean obtained for the surviving individuals at different times post-inoculation. Error bars represent the standard error of the mean.

Viral genome in blood was detectable in partridges from 1 to 11 dpi (Figure 3). However, viremia (measured by standard plaque-formation) could only be detected at 3 and 5 dpi (Figure 3B). Both viremia titres (1.8 × 10^5^ pfu/mL) and viral genome load peaked at 3 dpi. In blood samples with a Ct > 34, the virus was not detected by standard plaque formation, which indeed confirms a higher sensitivity of the real time RT-PCR assay used in this study over plaque assay for virus detection in blood.

In the house sparrows, blood samples collected at 1, 3, 5, 7 and 9 dpi, were negative for BAGV viral genome assessed by real time RT-PCR, and the same result was observed in blood samples from mice collected at 3 dpi (Table 1 and data not shown).

### Serology

**Figure 4:**
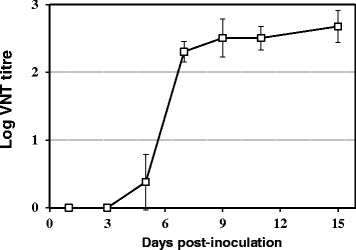
**Antibody response to BAGV in serum from BAGV-inoculated red-legged partridges.** The course of the antibody response in the individuals inoculated with BAGV, measured on different days post-inoculation, is plotted as a solid line with open squares representing the mean log titres of BAGV-neutralizing antibodies measured in the virus-neutralization test (VNT). Error bars represent the standard deviation of the data.

BAGV-neutralizing antibodies (NtAb) were observed in all partridges surviving the infection. NtAb were first detected at 5 dpi and were observed in all the partridges by 7 dpi with titres ranging from 1:160 to 1:320 and reaching maximum titres (up to 1:1280) at the end of the experiment (Figure 4 and Table 1). In contrast, NtAb were absent from the blood of the inoculated house sparrows at 15 dpi (Table 1). As for the mice, only those inoculated with the highest dose (5 × 10^5^ pfu) developed detectable NtAb at 21 dpi, showing low titres (1:10 to 1:20), the rest being negative (titre <1:10).

### Virus shedding and viral load in feathers

**Figure 5:**
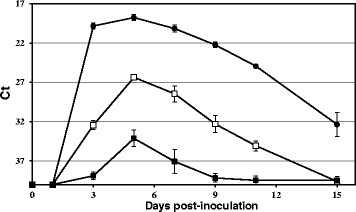
**Viral genome load in swabs (oral, cloacal) and feathers from BAGV-inoculated red-legged partridges.** The course of the viral RNA load in each type of sample, measured by real-time RT-PCR specific for BAGV, is represented on different days post-inoculation. Open squares indicate viral load in oral swabs; closed squares viral load in cloacal swabs and closed circles viral load in feathers. Error bars represent standard error of the mean.

In the inoculated red-legged partridges, virus shedding was shown to occur from 3 to 11 dpi through the cloacal and oral routes, reaching a maximum at 5 dpi, but it was more consistently detected in oropharyngeal than in cloacal swabs (Figure 5).

The viral genome load in feathers reached a peak also at 5 dpi, but it was found to be considerably higher and was detectable for a longer period than in blood and in both types of swabs examined. At 15 dpi, the virus genome was still detectable in feathers, while in the swabs, no virus genome was observed beyond 11 dpi.

### Virus distribution in organs

The post-mortem examination of the lethally infected red-legged partridges revealed systemic infection, as the virus was detected by real-time RT-PCR in all organs examined, including the brain, heart, spleen, kidney and liver (Table 2). In order to assess the extent of virus distribution in organs at different times post-infection, viral loads were compared in organs collected from BAGV-inoculated birds subjected to programmed necropsies at 4, 7 and 10 dpi and from the birds surviving the infection after 15 dpi. While in the heart, kidney, spleen and liver, the highest viral loads were found at 4 dpi, that is, only 1 day after the viremia peak, the maximum viral load in the brain was observed 3–5 days later, that is, at 7–10 dpi. After that, over time, the viral genome load declined, but at day 15 post infection, the virus could still be detected in most organs (see Additional file 1).

**Table 2:** **BAGV detection by real time RT-PCR in organs from inoculated red-legged partridges**

**Partridge**	**Sex**	**dpi**	**Viral load in organs (Ct)**				
			**Brain**	**Heart**	**Kidney**	**Spleen**	**Liver**
N 1	female	4	29	19	21	21	21
N 4	male	4	30	25	23	23	22
4*	female	6	27	23	23	23	24
N 2	female	7	22	22	22	24	23
N 5	male	7	27	23	25	27	30
10*	male	7	24	21	25	24	25
N 3	female	10	30	29	27	32	31
N 6	male	10	26	28	23	29	33
7*	male	10	21^†^	26	25	28	33
1	male	15	34	>40	37	34	>40
2	female	15	31	32	31	29	37
3	female	15	33	32	32	32	37
5	female	15	31	32	32	31	34
6	male	15	33	35	34	30	38
8	male	15	31	32	30	30	38
9	male	15	35	35	34	31	35

Individual partridges are numbered correlatively (N indicates partridges in the programmed necropsy group). The table indicates the sex of the sampled partridges, the day post infection (dpi) when the samples were obtained and the corresponding viral load expressed in Ct (threshold cycle) obtained by real-time RT-PCR analysis, for the different organs examined.

*Lethally infected birds; ^†^Complete sequence analyzed.

One of the tissue samples examined (the brain from the necropsy of partridge 7, which died at 10 dpi) which showed a high viral genome load (Ct = 21) (Table 2) was chosen for further genetic characterization by complete sequence analysis. The sequence obtained (GenBank:KR108246), was identical to the complete sequence of the BAGV strain used for the inoculation (GenBank:KR108245).

### Contact transmission in red-legged partridges

None of the four contact partridges died during the experiment. However, all of them showed viremia, viral shedding through the oral and cloacal routes and the presence of virus in feathers. Furthermore, three of them suffered important body weight loss and developed neutralizing antibodies. The presence of viral RNA was confirmed in all analyzed tissues at the end of the experiment (Table 3).

**Table 3:** **Summary of the main results obtained in contact partridges**

	**Contact 1**					**Contact 2**					**Contact 3**					**Contact 4**				
dpi	7	9	11	15	18	7	9	11	15	18	7	9	11	15	18	7	9	11	15	18
% init. weight	96	94	91	81	83	98	97	98	100	96	98	97	97	91	76	99	99	100	90	91
VNT titre	<10	<10	20	640	320	<10	<10	<10	<10	<10	<10	<10	<10	80	320	<10	<10	<10	160	320
VL blood (Ct)	29	30	32	39	38	>40	>40	>40	26	29	>40	>40	25	33	38	>40	25	20	33	35
VL oral. (Ct)	37	28	24	29	>40	>40	>40	>40	>40	25	33	>40	33	28	31	>40	33	28	28	36
VL cloacal (Ct)	>40	>40	>40	>40	>40	>40	>40	>40	>40	28	>40	>40	>40	37	>40	>40	>40	37	>40	36
VL feather (Ct)	34	20	20	24	37	>40	>40	>40	25	20	>40	>40	23	21	23	>40	35	19	22	24
	Contact 1					Contact 2					Contact 3					Contact 4				
VL in organs (18 dpi)	brain	heart	kidney	spleen	liver	brain	heart	kidney	spleen	liver	brain	heart	kidney	spleen	liver	brain	heart	kidney	spleen	liver
	32	33	32	34	36	19	19	20	25	21	23	32	25	22	33	28	26	26	29	32

The upper part of the table shows the values obtained from the four contact partridges on different days post-inoculation (dpi) for the following parameters: percentage of weight compared to initial weight at day 0 (% init. weight); neutralizing antibody titre in serum measured by the virus neutralization test (VNT titre); viral load (VL) in blood, oral and cloacal swabs and in feathers, as measured by real time RT-PCR, expressed in Ct values. The bottom part of the table shows the observed viral load (VL) measured by real-time RT-PCR (expressed in Ct values) in five different organs (the brain, heart, kidney, spleen and liver) at the time of necropsy of the contact partridges (18 dpi) (neg = Ct ≥40).

Three out of four contact partridges showed a delay of 5 to 9 days in the development of disease signs and evidence of infection (viral load, antibodies in serum and weight loss), as compared to inoculated birds (Table 3). In one of these contact partridges (contact 3), a viral load in oral swab was detected four days before it became viremic. In the fourth contact partridge, signs of the disease and evidence of infection appeared approximately 13 days later than in the inoculated group.

## Discussion

To our knowledge, this study is the first one describing an experimental infection with BAGV in a European wild bird species, the red-legged partridge. In laboratory-controlled conditions, the study demonstrated that this species is susceptible to BAGV infection and, as a consequence, develops a disease with signs compatible with those observed in field conditions in the outbreak in Cádiz, Spain, in 2010 [13]. Therefore, Bagaza virus was concluded to be the causative agent of this disease. The virus appeared to be rather stable as its complete genome sequence, obtained from the brain of one of the clinically affected partridges, was identical to the genome sequence of the virus strain used in the inoculum. In contrast, under the experimental conditions assayed, the virus affected neither house sparrows nor mice, which presumably reflects some sort of resistance to the infection in these species that can rely on their genetic background and/or innate immune mechanisms. A role for adaptive (humoral) immunity was specifically discarded, since all the animals were found to be negative for specific BAGV antibodies prior to inoculation.

In the outbreak in Southern Spain in 2010, BAGV not only affected red-legged partridges but also two other wild bird species: the common pheasant (*Phasianus colchicus*) and, to a lesser extent, the common wood pigeon (*Columba palumbus*) [13]. Other avian species, such as the domestic turkey (*Melleagris gallipavo*) or the Japanese quail (*Coturnix coturnix*), were proven susceptible to natural and experimental infection caused by old strains of the synonymous virus ITV [16,22,23] whereas chickens (except one-day-old chicks), ducks and pigeons were not susceptible [23]. Therefore, the host range of BAGV seems to mainly include members of the *Phasianidae* family, comprising partridges, turkeys, pheasants, quails and chickens. This study further supports this host range, since red-legged partridges, but not house sparrows, where shown susceptible to the disease. It is worth noting that the BAGV host range differs in this regard from that of WNV, which includes passerines like the house sparrow [24,25].

Regarding mammals, suckling mice had been shown susceptible to infection and disease after experimental inoculation of an Indian BAGV strain using the *Culex tritaniorhynchus* mosquito as the vector [17]. Experiments performed with an old ITV strain (1960) showed that it could be transmitted (although at low rates) to suckling mice by *A. aegyptii* and *Culex pipiens* bites. In adult mice, ITV inoculation caused mortality when inoculated by intracerebral or intranasal routes but not when intraperitoneal or subcutaneous routes were used. This strain did not produce illness in adult hamsters or guinea pigs after intracranial inoculation [23,26]. In keeping with these previous observations, our results further suggested that BAGV has little effect, if any, on intraperitoneally inoculated adult mice.

Clinical signs observed in red-legged partridges during the experiment (weakness, reluctance to move, lack of avoidance to capture, and partially closed eyes) correlated well with neurologic signs and apparent blindness registered in natural infections [13,14]. However, the observations made under experimental conditions might not completely mimic what occurs in nature. The mortality rate in the inoculated partridges was 30%, but morbidity reached 100%, as all inoculated birds suffered significant weight loss. It is very likely that individuals that survived the experimental infection but suffered clinical illness would probably have died in the wild as a result of difficulties with feeding and/or escaping from predators. Viral pathogenicity evaluation in experimental trials is usually based on survival rates. Nevertheless, when experimental infections are performed in wild birds, the number of individuals is often limited due to animal welfare restrictions and logistic constraints derived from the wild origin of birds [27]. Consequently, statistically significant differences in mortality rates between groups are frequently difficult to observe. In this study, we evaluated not only mortality rates but also a morbidity index i.e. body weight loss, which proved to be a good indicator of disease severity and revealed dramatic differences between inoculated and control groups during the acute phase of the infection. This observation suggests that the accurate evaluation of quantitative indexes of morbidity, such as weight, can be useful to properly evaluate the pathogenicity of virus strains in an experimental setting.

Based on the data obtained on viremia, viral load in blood, oral and cloacal swabs, and feathers, the course of BAGV infection was similar to what has been described for the West Nile virus in partridges and corvids [28-30]. The delayed course of the viral load observed in the brain with respect to other peripheral organs suggested that crossing the blood–brain barrier is a late event during the infection. Persistent infections by other flaviviruses have been involved in alternative modes of transmission by predation of the affected avian hosts [31]. In our study, the BAGV genome was present in organs and feathers up to 15 dpi, but to properly assess this point, it would be necessary to carry out more prolonged experiments with higher number of birds. Despite similarities found in neutralizing antibody responses of red-legged partridges infected with BAGV and WNV [29], antibodies appeared slightly earlier (at 7 dpi) and reached higher titres at 15 dpi of BAGV infection as compared to the WNV infection.

In this work, female partridges showed greater and more prolonged body weight loss after infection than males. A greater impact on female pheasants was also reported during the outbreak of BAGV in Spain, causing a reduction from 4 to 2.4 in the female/male ratio of hunted pheasants during the following hunting season [13]. Also in South Africa, a higher impact of illness in female turkeys was registered for the synonymous ITV [22]. However, the reason for this difference is still unclear.

BAGV-inoculated partridges showed a viremia peak higher than 10^5^ pfu/mL. The viremia level necessary for BAGV infection of feeding mosquitoes has not been determined, but 10^4^-10^5^ pfu/mL is the threshold of infectious viremia that has been experimentally established for other flaviviruses such as WNV to transmit the infection to *Culex* mosquitoes, one of its competent vectors [32,33]. If a similar threshold is assumed for BAGV, then the red-legged partridge could be considered a moderately competent host for BAGV transmission by mosquito bite, and therefore this species could play a role as a reservoir or amplifying host in nature. Viral shedding was higher and more consistent in oral secretions than in faeces, which is in accordance with the data obtained in field samples from partridges and pheasants during the BAGV outbreak [13]. Viral RNA was detected in feathers of infected birds at higher rates and for a longer period after infection in comparison with other samples (blood and swabs), so the feather could be a convenient sample to monitor virus circulation in surveillance programs, at least in red-legged partridges. A much greater viral load in growing feathers and with a longer persistence than in blood has also been found for Eastern equine encephalitis virus in ring-necked pheasants, avian leucosis virus in domestic chickens and West Nile virus in Gyr-Saker hybrid falcons [34,35]. Viral detection in vascular and non-vascular feathers of corvid carcasses has been proposed as useful for West Nile virus surveillance [34,36].

Interestingly, in this study, all contact-exposed birds became viremic and seroconverted, showing a high rate of contact transmission of BAGV in this host. In order to remove any potential ectoparasite present, all the individuals had been treated with pyrethroids before entering the experimental setting at the BSL3 facilities; consequently only direct transmission could explain the infection of contact individuals. In three out of four contact partridges, the signs of the disease started with a delay of 5 to 9 days with respect to syringe-inoculated birds. The high amount of virus present in oral secretions and feathers of syringe-inoculated birds at that point suggests that the infection in contact-exposed birds was probably acquired through oral-oral route or by skin or feather picking. Viral detection in the oral swab in a contact partridge 4 days before viremia supports the idea of infection by the oral route. In the fourth contact partridge, the disease symptoms started 13 days later than in the syringe-inoculated partridges, suggesting that this partridge could have acquired the infection from the other contact birds in a second round of contact infection, and not from the inoculated group. This result contrasts with our previous observation that WNV was not transmitted to contact red-legged partridges under analogous experimental conditions [29]. Potential direct transmission of WNV has been assessed experimentally for at least 24 avian species [27], but contact transmission has only been demonstrated in five species belonging to three families: Common goose (*Anser anser*) [37], ring-billed gull (*Larus delawarensis*) and three species of corvids [33]. Nevertheless, the contact transmission rate observed in this work (4 out of 4 contact birds) for BAGV is considerably higher than that registered for WNV in any of the studied species.

Although contact transmission has been proposed to explain the rapid dissemination of ITV strains in turkey farms [22], this could not be proved in experimental trials and intranasal infection attempts in turkeys were also unsuccessful [16]. The high transmission rate observed in the present study suggests that partridges are highly competent hosts for BAGV contact transmission, and, at least in this avian species, this transmission route can be epidemiologically relevant. The red-legged partridge is one of the most important small game bird species in Southern Europe and the UK, where restocking of farm-bred birds for hunting purposes is common. In breeding farms and, to a lesser extent, in the hunting properties where game birds aggregate in high densities around artificial feeders and water points, transmission risk by direct contact through water/food contamination or feather picking could be high.

The fact that BAGV has appeared in Southern Europe just once indicates that its introduction in this continent is a very unusual event. The BAGV geographic range includes Sub-Saharan Africa, and consequently, this area could be a possible origin for the introduction of BAGV into Spain. This hypothesis, however, needs to be substantiated, for instance, with sequence data recent African BAGV strains, but also with a suitable mechanism of introduction. Viraemic migratory birds or wind-borne infected mosquitoes have been proposed as mechanisms mediating the introduction of other flaviviruses [38,39]. Nevertheless, other alternative explanations like poultry industry-driven movements or trading of exotic birds should not be disregarded. Evidence of BAGV circulation in Southern Spain in two consecutive seasons [6] supports the possibility of overwintering after the introduction of the virus and indicates a risk of expansion in areas with similar climate conditions and/or similar game bird release programs. Other epornitic arboviruses, such as WNV and USUV have spread slowly but relentlessly throughout large parts of Europe [1-4]. BAGV is likely to have a similar potential for dissemination, but displaying a more efficient contact transmission, at least in red-legged partridges, a characteristic that might cause large outbreaks in dense populations of susceptible birds, such as in farmed game birds, which could facilitate the adaptation and maintenance of the BAGV life cycle after a new introduction of the virus, and eventually the spread of this pathogen when game birds are released into the wild. Thus, epidemiological surveillance in these susceptible birds is needed in order to avoid the introduction, maintenance and spread of the disease.

In other European countries, other birds from the *Phasianidae* family (grey partridges, pheasants) play the same role for game as the red-legged partridge, being bred in captivity and released into the wild for hunting purposes. Consequently, susceptibility studies on these species are necessary to estimate to what extent the emergence of BAGV could affect game birds on the continent.

The emergence of BAGV in Spain has had dramatic consequences on game bird populations, in which it has caused high mortality rates, mainly in the red-legged partridge [5], a species with great economic and ecological value in Europe. Besides its economic importance, the risk of BAGV for public health is still unknown. On the one hand, its synonymous virus, ITV, has never been reported to cause illness in humans, despite circulating in turkeys in Israel for decades [23,40]; and on the other hand, the presence of neutralizing Ab against BAGV in humans with encephalitis suggests infection but not necessarily pathogenicity [11].

In summary, this study confirms that the red-legged partridge is susceptible to BAGV infection and disease, and can be a competent host for both mosquito and direct (contact) transmission. Consequently, the red-legged partridge constitutes a suitable target species for BAGV surveillance. Finally, feather sampling could be a useful strategy in active surveillance programs for early detection of BAGV.

## Additional file

Additional file 1:
**BAGV RNA load in organs from inoculated red-legged partridges.** Viral RNA load, expressed as threshold value (Ct) was measured in five different organs (the heart, spleen, kidney, brain and liver) of BAGV-inoculated red-legged partridges. Closed circles indicate viral load in partridges of the programmed necropsy group and open circles show viral load in lethally infected partridges. A solid line represents the mean of the data of viral load at different days post-infection.
