# Supplementary figures and images for: Bagaza virus is pathogenic and transmitted by direct contact in experimentally infected partridges, but is not infectious in house sparrows and adult mice
Source: Vet Res. 2015 Sep 4;46(1):93. doi: 10.1186/s13567-015-0233-9 (PMC4559182; doi:10.1186/s13567-015-0233-9)

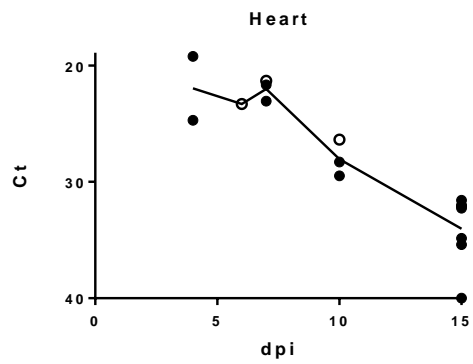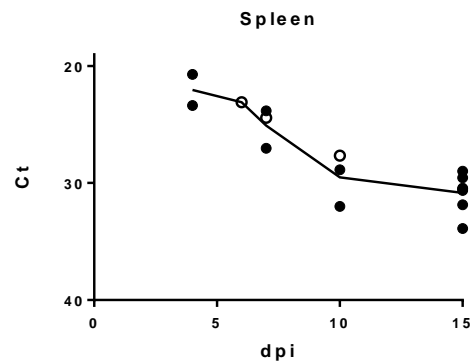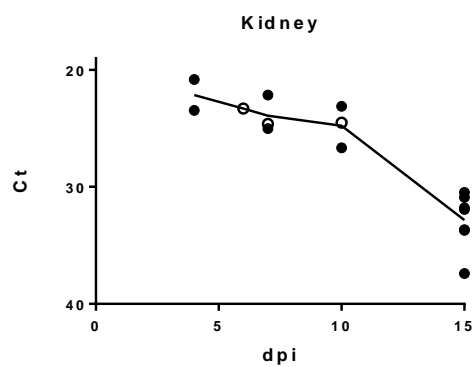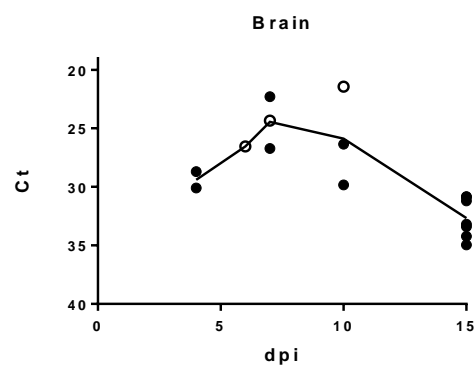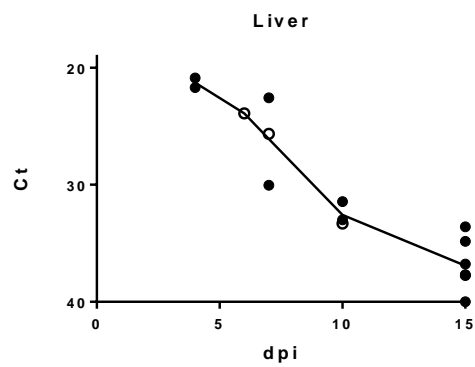

Supplement: Additional file 1: — BAGV RNA load in organs from inoculated red-legged partridges. Viral RNA load, expressed as threshold value (Ct) was measured in five different organs (the heart, spleen, kidney, brain and liver) of BAGV-inoculated red-legged partridges. Closed circles indicate viral load in partridges of the programmed necropsy group and open circles show viral load in lethally infected partridges. A solid line represents the mean of the data of viral load at different days post-infection. [file 13567_2015_233_MOESM1_ESM.pdf]
